# Incidence and risk factors of rhegmatogenous retinal detachment following paediatric cataract surgery: A systematic review and meta‐analysis

**DOI:** 10.1111/aos.17533

**Published:** 2025-06-04

**Authors:** Hashem Abu Serhan, Saad Ashraf, Ayesha Shaukat, Ajeet Singh, Hasnaa Abdelrhem, Hamza Irfan, Fariha Arif, Abdullah Ahmed

**Affiliations:** ^1^ Department of Ophthalmology Hamad Medical Corporation Doha Qatar; ^2^ Department of Medicine Dow University of Health Sciences Karachi Pakistan; ^3^ Department of Chemistry, Faculty of Science Cairo University Cairo Egypt; ^4^ Department of Ophthalmology Shaikh Khalifa Bin Zayed Al Nahyan Medical and Dental College Lahore Pakistan; ^5^ Department of Medicine Allama Iqbal Medical College Lahore Pakistan

**Keywords:** congenital cataract, intraocular lens implantation, mental retardation, paediatric cataract surgery, rhegmatogenous retinal detachment, risk factors

## Abstract

To determine the incidence and identify risk factors of rhegmatogenous retinal detachment (RRD) following paediatric cataract surgery. This systematic review and meta‐analysis adhered to PRISMA guidelines and was registered in PROSPERO (CRD42024538383). A comprehensive search was conducted across multiple databases, including Cochrane CENTRAL, PubMed/MEDLINE, SCOPUS, Web of Science, ScienceDirect and Google Scholar, up to December 2024. Studies were included if they reported on RRD following cataract surgery in paediatric populations (0–18 years) with a minimum follow‐up of 4 months. Data extraction was performed independently by two authors, with disagreements resolved through consultation with a third author. Risk of bias was assessed using the Newcastle‐Ottawa Scale, and statistical analysis was performed using R software version 4.5.0, with leave‐one‐out analysis conducted for outcomes with substantial heterogeneity (*I* ^2^ > 50%). The meta‐analysis included data from 5922 eyes across nine studies. The mean age of the participants was 7.08 ± 3.13 years, with a mean follow‐up duration of 4.02 years. The pooled incidence of RRD after paediatric cataract surgery was 2.4% [95% CI: 0.7%; 5.0%], with significant heterogeneity observed (*I* ^2^ = 85.8%). Patients without primary intraocular lens (IOL) implantation exhibited a numerically higher incidence of RRD (6.1% [95% CI: 0.6%; 15.9%]) compared to those with IOL implantation (1.9% [95% CI: 0.0%; 6.8%]); however, this difference was not found to be statistically significant (*p* = 0.2667). Similarly, the incidence of RRD was 1.0% [95% CI: 0.3%; 2.2%] in unilaterally operated eyes and numerically higher at 2.1% [95% CI: 1.0%; 3.6%] in bilaterally operated eyes, though this difference was also not statistically significant (*p* = 0.2563). Children with mental retardation demonstrated a significantly higher risk of RRD, with an incidence of 11.0% [95% CI: 6.6%; 16.4%]. Paediatric cataract surgery carries a notable risk of rhegmatogenous retinal detachment, with significant variability in incidence across different patient populations. While numerically higher incidences of RRD were observed in patients without primary IOL implantation and in bilaterally operated eyes compared to their counterparts, these differences did not reach statistical significance in this meta‐analysis. The presence of mental retardation was identified as a significant risk factor. Enhanced postoperative monitoring and individualised surgical approaches are recommended, particularly for high‐risk groups. Future research should focus on prospective studies with standardised protocols and longer follow‐up periods to better understand causal relationships and refine preventive strategies.

## 1 INTRODUCTION

Childhood vision impairment represents a significant global health challenge, with cataracts being one of the principal causes (Sheeladevi et al., 2016). Paediatric cataracts can be classified as congenital (present at birth) or developmental (appearing during childhood) (Khokhar et al., 2017). The overall prevalence of childhood cataract ranges from 0.32 to 22.9 per 10 000 children globally (median 1.03/10000), while congenital cataracts specifically have a reported prevalence of 0.63 to 9.74 per 10 000 (median 1.71/10000) (Sheeladevi et al., 2016). Significant regional variations exist; prevalence estimates for childhood cataract range from 0.42 to 2.05 per 10 000 in low‐income economies and 0.63 to 13.6 per 10 000 in high‐income economies, based on the studies reviewed by Sheeladevi et al. (2016). By contrast, developmental cataracts, which emerge after birth, often during the first decade of life, have a reported incidence of 1.8 to 3.6 per 10 000 children annually (Sheeladevi et al., 2016).

Untreated paediatric cataracts cause significant visual impairment, accounting for 7.4% to 15.3% of childhood blindness worldwide (Gupta et al., 2025). Without timely intervention, these cases often lead to amblyopia, nystagmus and poor fixation (Gupta et al., 2025). Early surgical intervention is therefore crucial for visual rehabilitation and has profound implications for an affected child's personal, social and educational development (Khokhar et al., 2017).

The surgical management of paediatric cataracts presents unique challenges compared with adult procedures. Various technical difficulties arise from low scleral rigidity, smaller eyeball size, shallow anterior chamber depth, small pupil size, an elastic anterior capsule, high positive intravitreal pressure and an increased risk of vitreous loss (Gupta et al., 2025). Current evidence suggests that bilateral visually significant cataracts should be removed between 6 and 8 weeks of age, while unilateral cases should be addressed between 4 and 6 weeks (Gupta et al., 2025). However, balancing the need for early intervention against the risk of complications requires careful consideration (Nyström et al., 2020).

Paediatric cataract surgery techniques have evolved significantly over recent decades. Anterior capsulorhexis is typically performed using manual continuous curvilinear capsulorhexis (CCC) (Khokhar et al., 2017), while femtosecond laser‐assisted approaches are also utilised in specialised centres (Vasavada, 2018). To prevent visual axis opacification (VAO), a significant postoperative complication, primary posterior capsulotomy combined with anterior vitrectomy is routinely performed, particularly in younger patients (<6 years of age) (Khokhar et al., 2017).

The management of paediatric cataracts has been extensively documented through registries such as the Paediatric Cataract Register (PECARE), which tracks outcomes in Sweden and Denmark (Magnusson et al., 2018). According to data from PECARE, primary intraocular lens (IOL) implantation was performed in 71.6% of paediatric cataract surgeries between 2007 and 2013, indicating widespread adoption of this approach (Magnusson et al., 2018). While primary IOL implantation became common practice for many paediatric cataract surgeons, including for infants (Magnusson et al., 2018; Solebo et al., 2018), some centres maintained a more cautious approach, particularly for very young infants (e.g. <12 weeks) or those with specific ocular characteristics like axial length <15 mm, sometimes opting for aphakia and later IOL implantation (Nyström et al., 2020). This cautious stance is supported by findings from studies like the IoLunder2 cohort study, which indicated that primary IOL implantation in children under 2 years of age does not confer better vision or protection against postoperative glaucoma and significantly increases the risk of requiring early reoperation for visual axis opacification (Solebo et al., 2018).

Postoperative complications remain a significant concern in paediatric cataract surgery. Visual axis opacification (VAO) is a common postoperative complication; for instance, the IoLunder2 study reported that 48% of children under 2 years who received an IOL experienced VAO, often requiring reoperation (Solebo et al., 2018). Specialised IOL designs, such as the bag‐in‐the‐lens IOL, have been developed to mitigate this, with studies reporting significantly lower rates of VAO requiring treatment (around 4.6%) with this type of implant (Nyström et al., 2020).

Secondary glaucoma is another critical postoperative complication. Studies report varying incidences; for example, Nyström et al. (2020) found that 13.8% of eyes developed glaucoma in their cohort undergoing BIL‐IOL implantation, with a significantly higher risk (48.3%) in children operated on at ≤12 weeks of age (Nyström et al., 2020). The onset can be early, often within months of surgery (mean 15 weeks in the Nyström et al. 2020 cohort) (Nyström et al., 2020), though glaucoma can also manifest years later (Keech et al., 1989). Risk factors for secondary glaucoma include early age at surgery (particularly ≤12 weeks), presence of microcornea, and other ocular comorbidities or anterior segment abnormalities (Nyström et al., 2020).

Retinal detachment (RD) after cataract surgery is a rare but vision‐threatening complication that requires surgical intervention (Rabiah et al., 2005). Retinal detachments in the paediatric population are generally uncommon (Gan & Lam, 2018). In children, retinal detachment is recognised as a potential late complication of cataract surgery. While some cohorts have shown mean intervals to RD of around 6.8 years postsurgery, other historical reports involving older surgical techniques suggest mean intervals of 20 to 33 years (Rabiah et al., 2005).

Previous studies investigating retinal detachment after paediatric cataract surgery have often been characterised by relatively short‐term follow‐up (e.g. mean 3.7 to 6.8 years in some cohorts) or have focused predominantly on outcomes in aphakic eyes (Keech et al., 1989; Rabiah et al., 2005). Furthermore, while some studies have explored associations between certain surgical steps (like posterior capsulotomy, which was not found to be a predictor by Rabiah et al., 2005) and retinal detachment risk, the comprehensive impact of various modern surgical procedures and intraocular lens implantation strategies on the long‐term risk of detachment remains an area requiring further clarification (Rabiah et al., 2005). This systematic review and meta‐analysis aims to provide comprehensive, long‐term data on the incidence and risk factors of retinal detachment following paediatric cataract surgery, considering both aphakic and pseudophakic eyes, as well as various surgical techniques.

## 2 METHODS

This systematic review and meta‐analysis was performed in compliance with the Preferred Reporting Items for Systematic Reviews and Meta‐Analyses (PRISMA) guidelines (Page et al., 2021). The protocol of this systematic review and meta‐analysis was registered in PROSPERO with the reference number CRD42024538383.

### 2.1 Data sources and search strategy

A comprehensive search was conducted using the Cochrane CENTRAL, PubMed/MEDLINE, SCOPUS, Web of Science, ScienceDirect and Google Scholar databases from inception to July 2024. The reference lists of the retrieved articles and prior meta‐analyses were also screened for any relevant articles. The search strategy imposed no restrictions on publication status or language. The search terms included relevant PubMed MeSH terms and related keywords, such as (paediatric OR children OR infant* OR adolescent_) AND (cataract surgery OR cataract extraction OR phaco_) AND (retinal detachment OR retinal tears OR holes OR breaks). The detailed search strategy is provided in Table S1.

### 2.2 Study selection and eligibility criteria

All articles retrieved from the systematic search were imported into Rayyan software, where duplicates were subsequently removed (Ouzzani et al., 2016). Two authors (SA and AS) independently reviewed and selected studies, with any disagreements resolved by a third author (HAS). Selected studies were retrieved for full‐text review to confirm their relevance. The inclusion criteria for the studies were as follows: (1) studies that reported on retinal detachment following cataract surgery in paediatric populations (age range 0–18 years); (2) studies focusing on risk factors associated with retinal detachment postsurgery in this age group; (3) randomised trials and observational research, including cross‐sectional, case–control and cohort studies with a minimum follow‐up duration of 4 months; and (4) studies with clearly defined surgical techniques and outcomes that could be homogenised for analysis. However, reviews, editorials, case reports, letters, meta‐analyses, consensus reports, unavailable full‐text articles and studies lacking necessary information were excluded from consideration. The detailed inclusion/exclusion criteria of each included study are given in Table S2.

### 2.3 Data extraction

Two authors (SA and AS) conducted independent evaluations of the data and supplementary materials, resolving any discrepancies through consultation with a third author (HAS). The following data were extracted from the included studies: authorship, year of publication, study design and location, number of participants, patient's baseline characteristics, as well as outcomes related to the incidence and risk factors for retinal detachment. The extracted outcomes included details regarding the overall incidence of retinal detachment after cataract surgery, the incidence in patients with and without primary intraocular lens (IOL) implantation, the incidence in unilaterally and bilaterally operated eyes, and the incidence in children with mental retardation.

### 2.4 Quality assessment

Quality assessment was performed by two authors (SA and FA) using the 9‐point Newcastle‐Ottawa Scale (Ottawa Hospital Research Institute, n.d.). Each study was evaluated across eight domains: representativeness of the exposed group, ascertainment of exposure, selection of the non‐exposed group, demonstration that the outcome of interest was not present at the start of the study, comparability of cohorts, outcome assessment, sufficiency of follow‐up time and adequacy of cohort follow‐up. A total score of ≥6 points was considered indicative of high quality. In instances of disagreement, a third author (HAS) was consulted to resolve the issue.

### 2.5 Statistical analysis

Statistical analysis was conducted using R software version 4.2.2. Proportions were pooled using the Freeman‐Tukey double arcsine transformation to stabilise variances, particularly for proportions close to 0 or 1, ensuring more reliable estimates. A random‐effects model was employed to account for potential heterogeneity across studies, as it assumes variability in true effect sizes due to differences in study design, populations or other factors. Heterogeneity was quantitatively assessed using the Higgins *I* ^*2*^ statistic, which describes the percentage of total variation across studies that is due to heterogeneity rather than chance. An *I* ^*2*^ value greater than 50% was considered indicative of substantial heterogeneity. For outcomes with substantial heterogeneity (*I* ^2^ > 50%), a leave‐one‐out (L1O) analysis was conducted to assess the influence of individual studies on the pooled estimates and to identify potential sources of heterogeneity. In cases where the number of eyes was not explicitly reported, the number of patients was used as a proxy for the number of events and sample size to maintain consistency in the analysis. To evaluate the potential for publication bias, funnel plots were created and visually inspected for asymmetry.

## 3 RESULTS

### 3.1 Literature search

**FIGURE 1:**
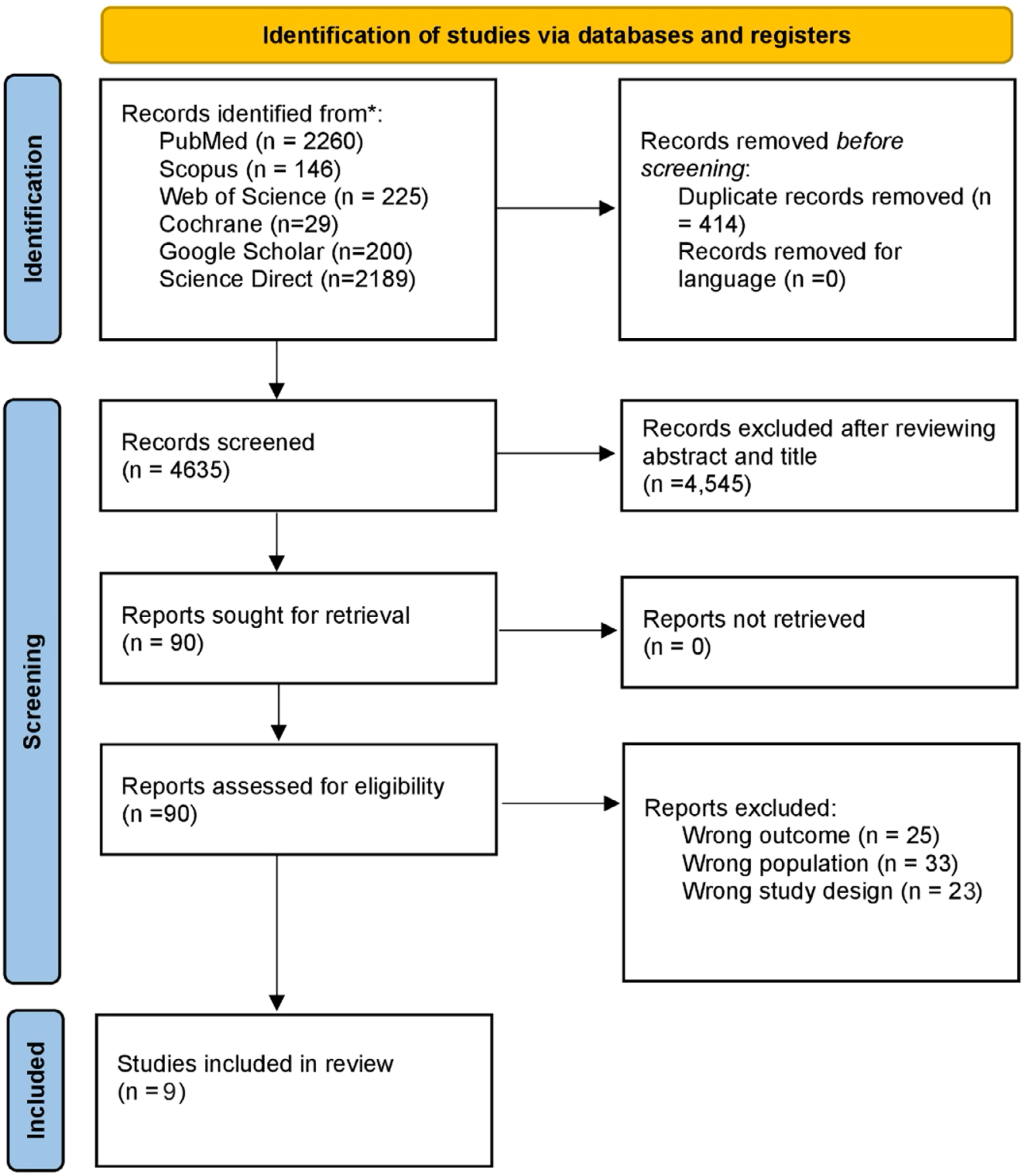
PRISMA flowchart of study selection process.

The preliminary literature search yielded 5049 results, which were screened for relevance based on titles and abstracts. After removing duplicates, 4635 records remained for further title/abstract screening, identifying 90 articles for potential full‐text review. Nine studies met the pre‐defined inclusion and exclusion criteria and were included in this systematic review (Agarkar et al., 2018; Giles et al., 2016; Haargaard et al., 2014; Koch et al., 2019; Ngoy et al., 2020; Oke et al., 2022; Rabiah et al., 2005; Sabr et al., 2024; Yen et al., 2023). The search and screening process is illustrated in the PRISMA flow chart Figure 1.

### 3.2 Baseline characteristics

This review includes a total of nine studies from various countries. Two of these studies were conducted in the USA (Oke et al., 2022; Yen et al., 2023) and Saudi Arabia (Rabiah et al., 2005; Sabr et al., 2024), while the others were distributed across different regions, including India (Agarkar et al., 2018), Cameroon (Giles et al., 2016), Denmark (Haargaard et al., 2014), Spain (Koch et al., 2019) and the Democratic Republic of the Congo (Ngoy et al., 2020). In total, 5788 patients were included, comprising a total of 6089 eyes analysed across different subgroups, with 2346 eyes belonging to males and 2165 eyes to females, respectively (note: the 5922 eyes for overall incidence is based on studies providing overall RD, while subgroup analyses like IOL or laterality may use slightly different total eye counts based on data availability for those specific stratifications). The mean age of the participants was 7.08 ± 3.13 years. The mean follow‐up duration was 4.02 years. The baseline characteristics are provided in Table 1.

**TABLE 1:** Baseline characteristics of all included studies.

Study	Year	Country	Total patients, *n*	Number of eyes (M/F)	Number of eyes, *n*	Mean age at cataract surgery, years (SD)	Follow‐up period, years (SD)	Primary IOL placement, *n* (% of eyes)	Number of eyes with retinal detachment, *n*	Indication for cataract surgery	Surgical procedure	Age at retinal detachment	Refractive status of eye	Pars plana approach/no pars plana approach	Final visual outcome
Sabr et al.	2024	Saudi Arabia	84	67/65	132	2.25	4 (3.7)	132 (100%)	20	Congenital cataract	Lensectomy with primary IOL implantation	Median of 7 years after cataract surgery (IQR 1 to 13 years)	Eyes with axial length ≤ 23.4 mm had a 0.55‐fold decreased odds of detachment. Preexisting myopia is a risk factor.	N/A	N/A
Yen et al.	2023	USA	491	261/230	609	5.6 (3.3)	5	609 (100%)	10	Paediatric cataract	Lensectomy with primary IOL implantation	N/A	N/A	N/A	N/A
Oke et al.	2022	USA	3289	1656/1633	3289*	5.1	N/A	2510 (76.3%)	47	N/A	N/A	Median time between initial surgery and RD repair was 47 days	N/A	480/2799	N/A
Ngoy et al.	2020	Democratic Republic of Congo	298	N/A	572	5.7 (4.3)	2.8 (0.9)	572 (100%)	3	Bilateral cataract	Standardised surgical treatment of paediatric cataract with IOL implantation	N/A	N/A	0/572	81.9% of patients had improved visual outcomes after surgery. Main reasons for reduced vision during follow‐up were secondary cataract (5.7%), IOL decentration (1.2%), retinal detachment (1.2%) and secondary glaucoma (1.5%).
Koch et al.	2020	Spain	46	31/25	56	2.8 (1.8)	10 (3.3)	56 (100%)	0	Paediatric cataracts	Lensectomy with primary IOL implantation	N/A	N/A	27/29	No incidences of retinal detachment reported.
Agarkar et al.	2017	India	295	298/183	481	12 (2)	5.5	481 (100%)	12	Congenital or developmental cataract (excluding traumatic cataracts, systemic anomalies and other ocular anomalies)	Lens aspiration through a scleral tunnel approach, primary posterior capsulorrhexis, automated anterior vitrectomy and primary intraocular lens implantation	Median 70 months after cataract surgery	Myopic (age‐adjusted ALD < −1 mm) and intellectual disability are risk factors.	0/481	5 eyes ≥6/18, and 9 eyes ≥6/60
Giles et al.	2016	Cameroon	50	33/29	62	10.2 (3.2)	0.3 (0.06)	54 (87.1%)	2	Congenital, developmental and traumatic cataract	Small incision cataract surgery (SICS) and manual phaco aspiration (MPA) with primary IOL implantation in most cases. Primary posterior capsulotomy was not routinely performed in children under 8.	N/A	N/A	0/62	Postoperative BCVA improved from 1.19 ± 0.93 to 0.58 ± 0.88 logMAR. Visual outcome was better in the developmental cataract group.
Haargaard et al.	2014	Denmark	656	N/A	1043	N/A	6.8	0 (0%)	25	Congenital cataract (excluding trauma, systemic or other ocular pathology).	Lensectomy, lensectomy with anterior vitrectomy or unspecified	Median of 9.1 years after cataract surgery	N/A	55/991	Three eyes went blind, one had hand movement, five eyes had VA <0.1, one eye VA 0.5, one eye VA <0.8.
Rabiah et al.	2005	Saudi Arabia	579	N/A	1017	≤16	6.8 (3.6)	0 (0%)	33	Cataract (excluding other ocular abnormalities except microcornea).	Limbal‐approach surgery without primary IOL implantation with a central posterior capsulotomy and limited anterior vitrectomy in some cases.	Mean 6.8 years postcataract surgery (median 7 years)	More myopic than the age‐adjusted aphakic norm is a risk factor.	N/A	N/A

Abbreviations: F, female; IOL, intraocular lens; M, male; SD, standard deviation; VA, visual acuity.

To explore the relationship between retinal detachment incidence and key patient and surgical variables across studies, Table 2 was created. For the ‘With Primary IOL Implantation’ analysis, four studies (Haargaard et al., 2014; Oke et al., 2022; Sabr et al., 2024; Yen et al., 2023) provided specific data. Haargaard et al. (2014) reported on eyes receiving primary IOL (0 RD events in 304 eyes) and those without primary IOL (25 RD events in 742 eyes). Sabr et al. (2024) also provided data for both patients with primary IOL (10 RD events in 94 eyes) and without (9 RD events in 34 eyes). Rabiah et al. (2005) exclusively included patients without primary IOL implantation. The ‘Without Primary IOL Implantation’ analysis included four studies that explicitly reported outcomes for patients without primary IOL implantation (Haargaard et al., 2014; Oke et al., 2022; Rabiah et al., 2005; Sabr et al., 2024). For the laterality analysis, three studies (Agarkar et al., 2018; Haargaard et al., 2014; Yen et al., 2023) provided data for both unilaterally and bilaterally operated eyes. Notably, in the Oke et al. (2022) study, the exact number of eyes was not specified for some stratifications, so the number of patients was used as a proxy where necessary.

**TABLE 2:** Relationship between retinal detachment incidence and key patient and surgical variables.

Variable	Number of studies reported	Contributing studies	Total number of eyes	Incidence rate of RD % [95% CI]
Overall incidence following cataract surgery	8	Sabr, Yen, Oke, Ngoy, Koch, Agarkar, Haargaard, Rabiah	5922	2.4% [0.7%; 5.0%]
Primary IOL status				
With primary IOL implantation	4	Sabr, Yen, Oke	3517	1.9% [0.0%; 10.3%]
Without primary IOL implantation	4	Oke, Giles, Haargaard, Rabiah	2572	6.1% [0.6%; 15.9%]
Laterality of surgery				
Unilaterally operated eyes	3	Yen, Agarkar, Haargaard	558	1.0% [0.3%; 2.2%]
Bilaterally operated eyes	3	Yen, Agarkar, Haargaard	1578	2.1% [1.0%; 3.6%]
Patient characteristics				
Children with mental retardation	2	Agarkar, Haargaard	173	11.0% [6.6%; 16.4%]

### 3.3 Quality assessment results

The risk of bias was assessed using the Newcastle‐Ottawa scale and is provided in Table S3. Agarkar et al. (2018) and Yen et al. (2023) scored the highest at eight, demonstrating a robust methodology with multicentre data and adequate follow‐up. Giles et al. (2016) scored the lowest at four, with limitations in representativeness and follow‐up. Koch et al. (2019) and Rabiah et al. (2005) scored six, reflecting moderate methodological strength. Haargaard et al. (2014), Ngoy et al. (2020), Oke et al. (2022) and Sabr et al. (2024) all scored five, sharing similar strengths and weaknesses in study design. The certainty of evidence using GRADE assessment is reported in Table 3.

**TABLE 3:** Summary of findings table including the assessment of the certainty of evidence through the use of the GRADE framework for reported outcomes in our review.

Outcomes	Category	Outcome endpoint	Studies (*N*)/eyes (*N*)	Estimate [95% CI]	Study limitations	Inconsistency (+/−)	Indirectness (+/−)	Imprecision (+/−)	Publication bias (+/−)	Certainty of evidence (GRADE)
Primary outcome [overall incidence of RD after paediatric cataract surgery]	Incidence of RD	Overall	8/5922	2.4% [0.7%; 5.0%]	Fair quality	Significant heterogeneity (−)	No concerns (+)	Wide CI	Funnel plot symmetry suggests no bias (+)	⊕⊕⊖⊖ Low
Secondary outcomes [risk factors for RD]	Surgical Characteristics	With IOL Implantation	4/3517	1.9% [0.0%; 6.7%]	Fair quality	Significant heterogeneity (−)	No concerns (+)	Wide CI	Funnel plot asymmetry suggests potential bias (−)	⊕⊖⊖⊖ Very Low
		Without IOL Implantation	4/2572	6.1% [0.6%; 15.9%]	Fair quality	Significant heterogeneity (−)	No concerns (+)	Wide CI	Funnel plot asymmetry suggests potential bias (−)	⊕⊖⊖⊖ Very Low
	Laterality of Surgery	Unilaterally Operated Eyes	3/558	1.0% [0.3%; 2.2%]	Fair quality	No heterogeneity (+)	No concerns (+)	Wide CI	Funnel plot symmetry suggests no bias (+)	⊕⊕⊖⊖ Low
		Bilaterally Operated Eyes	3/1578	2.1% [1.0%; 3.6%]	Fair quality	Moderate heterogeneity (−)	No concerns (+)	Wide CI	Funnel plot symmetry suggests no bias (+)	⊕⊕⊖⊖ Low
	Patient Characteristics	Children with Mental Retardation	2/173	11.0% [6.6%; 16.4%]	Fair quality	No heterogeneity (+)	No concerns (+)	Wide CI	Funnel plot asymmetry suggests potential bias (−)	⊕⊖⊖⊖ Very Low

### 3.4 Outcomes

#### 3.4.1 Overall incidence of retinal detachment after cataract surgery

**FIGURE 2:**
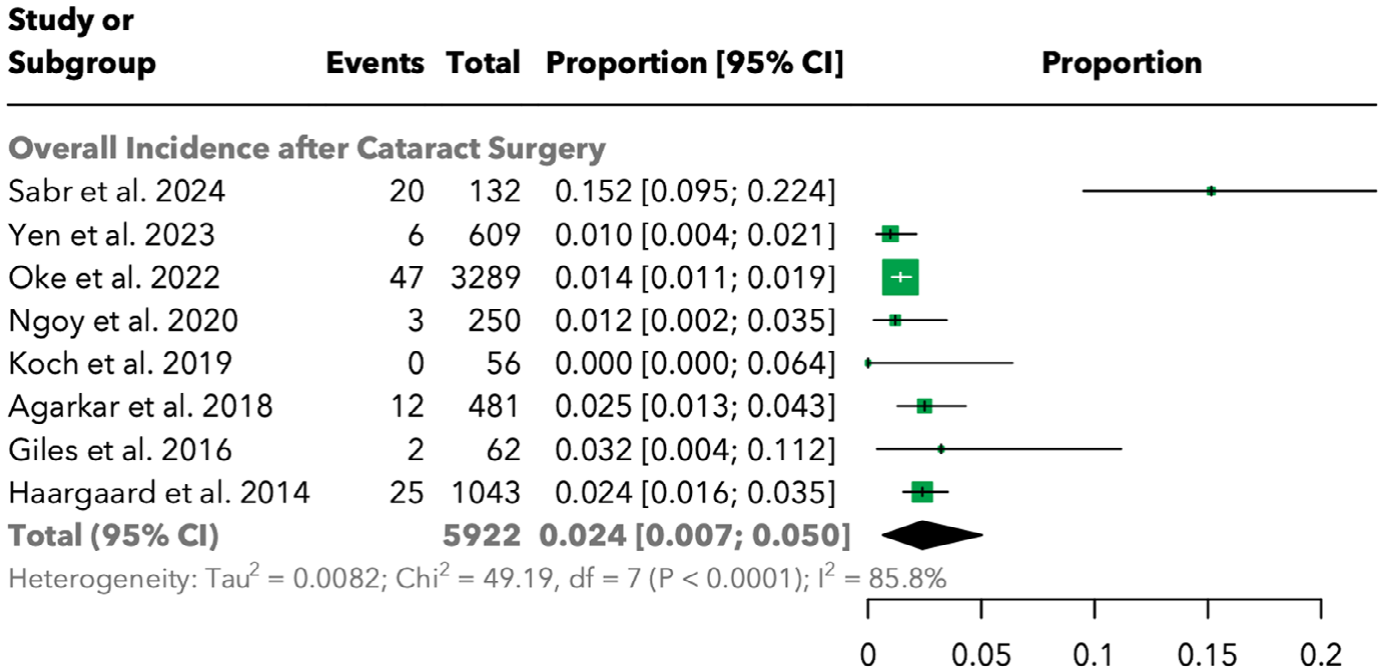
Forest plot of the overall incidence of retinal detachment (RD) after paediatric cataract surgery.

The overall incidence of retinal detachment (RD) following cataract surgery was analysed across multiple studies. The pooled data from 5922 cases revealed an overall incidence of 2.4% [95% CI: 0.7%; 5.0%]. Significant heterogeneity was observed among the studies (*I* ^2^ = 85.8%). The highest incidence was reported by Sabr et al. (2024) with 15.2% [9.5%; 22.4%], while the lowest was noted by Koch et al. (2019) with 0.0% [0.0%; 6.4%]. The detailed distribution of incidence rates across studies is illustrated in Figure 2.

#### 3.4.2 Incidence of retinal detachment after cataract surgery in patients with and without primary IOL implantation

**FIGURE 3:**
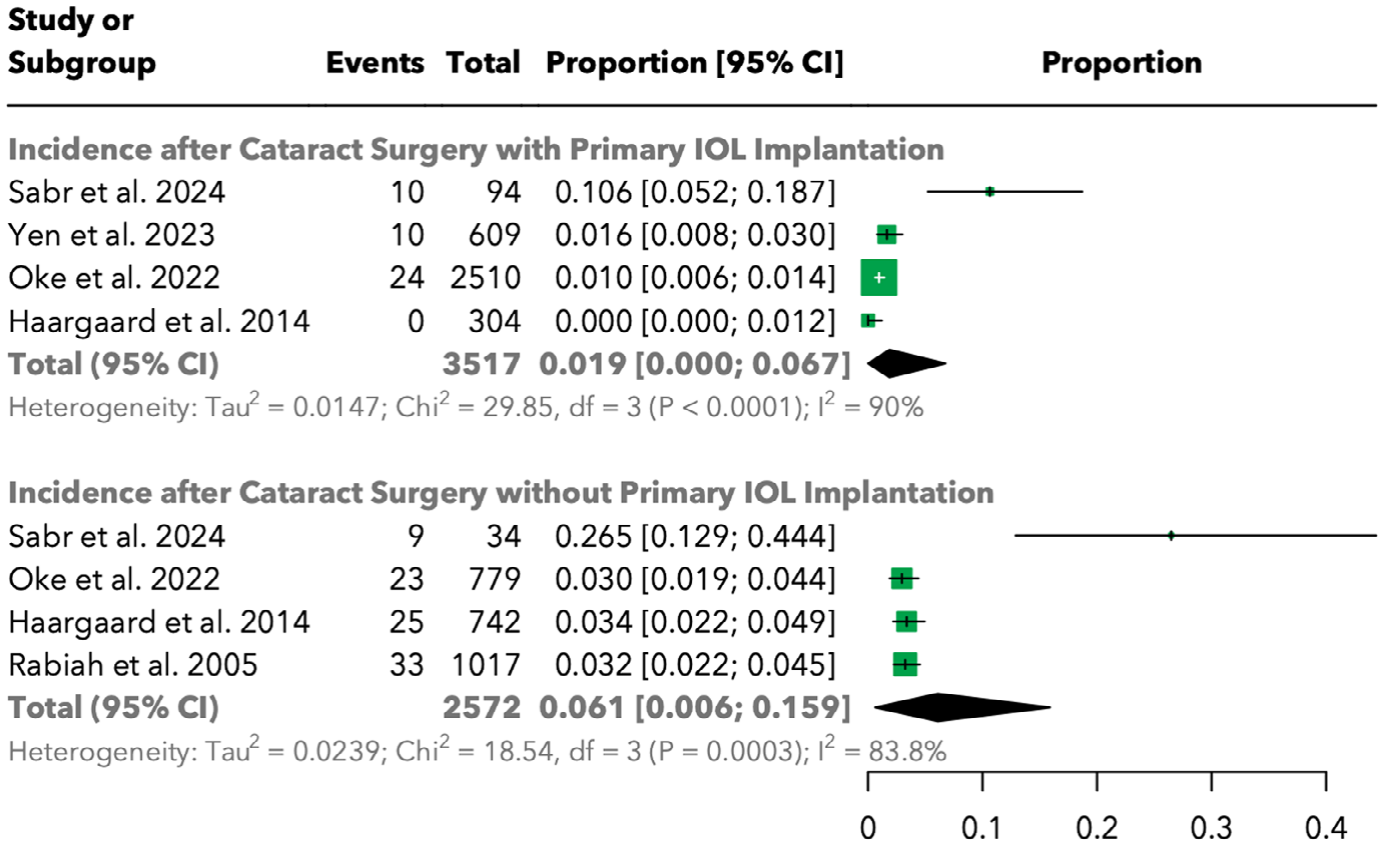
Forest plot comparing the effect of primary intraocular lens (IOL) implantation on the incidence of retinal detachment.

The incidence of RD was compared between patients who underwent cataract surgery with and without primary intraocular lens (IOL) implantation. In the group with primary IOL implantation, the total incidence was 1.9% [95% CI: 0.0%; 6.7%] across 3517 cases, with significant heterogeneity (*I* ^2^ = 90.0%). By contrast, the group without primary IOL implantation had a higher incidence of 6.1% [95% CI: 0.6%; 15.9%] across 2572 cases, also with significant heterogeneity (*I* ^2^ = 83.8%). This difference between both subgroups was not found to be statistically significant (*p* = 0.2667). The comparative analysis is presented in Figure 3.

#### 3.4.3 Incidence of retinal detachment after cataract surgery in unilaterally and bilaterally operated eyes

**FIGURE 4:**
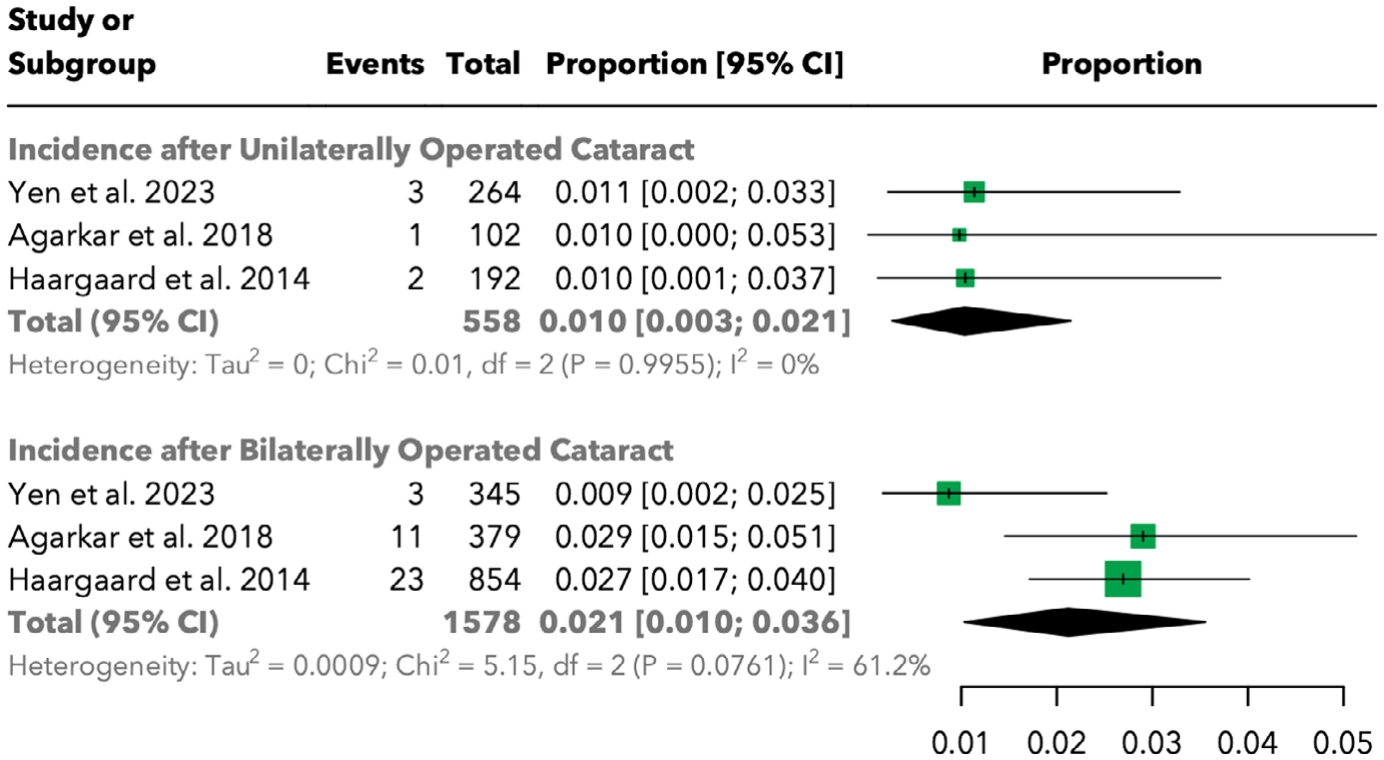
Forest plot of the incidence of retinal detachment in unilaterally and bilaterally operated eyes.

The incidence of RD was evaluated based on the laterality of cataract surgery. For unilaterally operated eyes, the total incidence was 1.0% [95% CI: 0.3%; 2.2%] across 558 cases, with no significant heterogeneity (*I* ^2^ = 0%). In bilaterally operated eyes, the incidence was numerically higher at 2.1% [95% CI: 1.0%; 3.6%] across 1578 cases, with moderate heterogeneity (*I* ^2^ = 61.2%). The difference in RD incidence between unilaterally and bilaterally operated eyes was not found to be statistically significant (*p* = 0.2563). It is important to note that while there appears to be a numerical trend suggesting a slightly elevated per‐eye risk in bilateral surgery compared with unilateral surgery, this meta‐analysis did not find sufficient evidence to conclude a statistically significant independent additional risk beyond simply having twice as many eyes at risk. The detailed results are shown in Figure 4.

#### 3.4.4 Incidence of retinal detachment after cataract surgery in children with mental retardation

**FIGURE 5:**
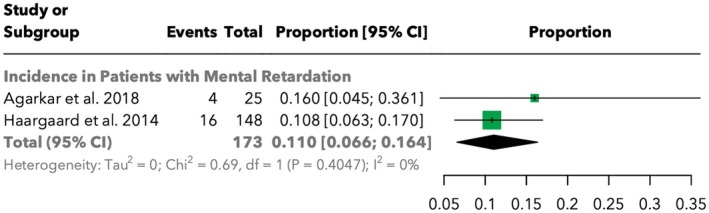
Forest plot of the incidence of retinal detachment in children with mental retardation.

The incidence of RD in children with mental retardation who underwent cataract surgery was also assessed. The pooled incidence was 11.0% [95% CI: 6.6%; 16.4%] across 173 cases, with no significant heterogeneity (*I* ^2^ = 0%). Agarkar et al. (2018) reported an incidence of 16.0% [4.5%; 36.1%], while Haargaard et al. (2014) reported 10.8% [6.3%; 17.0%]. The findings are summarised in Figure 5.

#### 3.4.5 Sensitivity analysis

A sensitivity analysis was conducted using two approaches (Table S4). First, we evaluated the impact of smaller studies by excluding those with fewer than 100 eyes (Koch et al., 2019 with 56 eyes and Giles et al., 2016 with 62 eyes). The pooled incidence of RD slightly increased to 2.8% [95% CI: 0.7%; 6.3%] across 5804 eyes, with heterogeneity remaining high (*I* ^2^ = 89.4%). Second, leave‐one‐out (L1O) analyses were performed for main outcomes. For overall RD incidence, omitting Sabr et al. (2024) reduced the pooled incidence to 0.4% [95% CI: 0.0%; 1.8%] with decreased heterogeneity (*I* ^2^ = 81.4%). When omitting Oke et al. (2022) or Haargaard et al. (2014), the pooled incidence increased to 2.9% [95% CI: 0.0%; 21.5%] and 4.2% [95% CI: 0.0%; 18.6%], respectively, with increased heterogeneity (*I* ^2^ > 95%).

For RD incidence with primary IOL implantation, omitting Sabr et al. (2024) yielded a pooled incidence of 0.7% [95% CI: 0.1%; 2.0%] with *I* ^2^ = 76.8%, while excluding other studies produced minimal changes. For RD without primary IOL implantation, omitting Sabr et al. (2024) resulted in a consistent estimate of 3.2% [95% CI: 2.5%; 3.9%] with no heterogeneity. For bilaterally operated eyes, omitting Yen et al. (2023) eliminated heterogeneity (*I* ^2^ = 0.0%) with a pooled incidence of 2.7% [95% CI: 1.9%; 3.7%]. These analyses confirm the robustness of our primary findings while highlighting the influence of the Sabr et al. (2024) study on overall estimates.

### 3.5 Publication bias

The assessment of publication bias using funnel plots revealed varying levels of symmetry across different outcomes (Table S5). For overall RD incidence, the funnel plot showed some asymmetry, with smaller studies reporting lower proportions of RD while one study with a high proportion also had a high standard error. Larger studies clustered around the pooled estimate, but the distribution suggested potential heterogeneity. For primary IOL status, slight asymmetry was observed, warranting cautious interpretation. The funnel plot for laterality of surgery appeared symmetrical, suggesting minimal publication bias. For RD in children with mental retardation, some asymmetry was noted, potentially indicating bias or systematic differences between studies. These findings highlight the importance of critical assessment when interpreting meta‐analytic conclusions, particularly for subgroups with observed asymmetry.

## 4 DISCUSSION

We conducted the first systematic review and meta‐analysis evaluating the incidence and risk factors of retinal detachment (RD) following paediatric cataract surgery. Our findings indicate an overall pooled incidence of 2.4% across 5922 cases, with significant variability across studies. Patients without primary intraocular lens (IOL) implantation showed a numerically higher incidence of RD (6.1%) compared to those with primary IOL implantation (1.9%), though this difference was not statistically significant (*p* = 0.2667). Similarly, unilateral surgeries demonstrated a numerically lower incidence (1.0%) compared with bilateral surgeries (2.1%), but again without statistical significance (*p* = 0.2563). Children with mental retardation had a significantly higher RD incidence (11.0%), suggesting that cognitive, genetic or behavioural factors may contribute to postsurgical complications in this group.

Our findings contrast with a previous study reporting a lower RD incidence of 3.22% (Giles et al., 2016). The larger sample size in our analysis strengthens the reliability of our estimate. The numerically lower RD incidence in eyes with IOL (1.9%) versus without IOL (6.1%) aligns with studies suggesting IOL implantation may provide protection against RD through improved vitreous stability (Inoue et al., 2005). However, this trend must be interpreted cautiously due to selection bias and lack of statistical significance. Historically, primary IOL implantation has been preferentially performed in older children and those with fewer ocular comorbidities, creating inherent differences between comparison groups.

The notably high RD incidence (15.2%) reported by Sabr et al. (2024) warrants examination. This may be attributed to their focus on congenital cataracts in a Saudi Arabian population with potentially unique genetic factors, a younger cohort (mean age 2.25 years) representing more severe cases, inclusion of patients with high myopia (a known RD risk factor) and more intensive follow‐up protocols potentially leading to higher detection rates.

The inclusion of the Oke et al. (2022) study was justified by its substantial contribution to the overall sample size, despite limitations in reporting eye‐specific data. The unusually short median time between surgery and RD (47 days) compared with other studies (typically years) might reflect differences in definition, follow‐up protocols or reporting patterns in claims data versus hospital‐based studies.

The higher RD incidence in children with mental retardation (approximately five times the overall rate) aligns with findings by Ngoy et al. (2020) and Haargaard et al. (2014), who reported a nearly 10‐fold increased risk (hazard ratio 9.59) in this population. This disparity likely stems from behavioural challenges affecting postoperative care compliance and increased ocular trauma risk, highlighting the need for specialised follow‐up protocols for this vulnerable group.

The risk of RD increases with age due to natural vitreous changes leading to vitreoretinal traction (Rosner et al., 1987). Additionally, myopic eyes are more prone to RD because eyeball elongation can cause retinal tears (Rosner et al., 1987). Comparing our paediatric findings with adult populations, the incidence of RRD within 1 year of cataract surgery in adults >40 years is approximately 0.21% (Morano et al., 2023), considerably lower than our paediatric rates. Both populations share common risk factors like myopia and surgical complexity, but paediatric cases involve additional risks from developmental and behavioural factors.

Several important risk factors warrant consideration, despite limited stratification in included studies. Glaucoma, with reported incidence rates of 2%–32% after paediatric cataract surgery (Zhang et al., 2023), may increase RD risk through altered ocular biomechanics, increased intraocular pressure causing vitreoretinal traction and additional surgical interventions. Multiple surgeries represent another important risk factor, as each intervention potentially disrupts the vitreous and creates inflammatory cascades contributing to retinal complications. Sabr et al. (2024) noted significantly higher RD risk in eyes with postoperative glaucoma (HR 49.01, *p* < 0.001) and inflammatory membranes (OR 6.75, *p* = 0.02), underscoring how complications requiring further intervention substantially elevate RD risk.

Our findings underscore the importance of careful patient selection, surgical planning and enhanced postoperative monitoring in paediatric cataract cases. Surgeons should consider identified risk factors when counselling parents and planning surgical approaches. The numerically higher incidence in aphakic eyes and bilaterally operated eyes, while not statistically significant, may still warrant consideration pending further research. Intraoperative techniques should minimise trauma and reduce postoperative complications, with careful consideration of IOL implantation benefits versus risks, especially in high‐risk groups.

This meta‐analysis offers several strengths, including rigorous methodology, comprehensive search strategy and extensive data stratification. However, limitations include variability in study designs and follow‐up durations introducing heterogeneity, potential selection and reporting biases in the retrospective studies, and geographical/demographic diversity that might limit applicability to specific contexts. We could not evaluate gender differences or specific refractive error types due to insufficient data from included studies. Future research should focus on prospective studies with standardised definitions and long‐term follow‐up to better understand causal relationships. Investigation of genetic factors and surgical technique advancements could refine risk stratification. Studies examining specific biological and environmental factors in high‐risk groups, particularly children with mental retardation, are needed. Long‐term cohort studies should explore RD's natural history and the impact of different surgical approaches, while technological advancements in imaging may enhance early detection and management.

## 5 CONCLUSION

In conclusion, this first systematic review and meta‐analysis of retinal detachment following paediatric cataract surgery provides crucial insights into its incidence and risk factors. Our analysis of nearly 6000 cases demonstrates that while the overall risk of retinal detachment is relatively low at 2.4%, certain patient groups face significantly higher risks. Particularly concerning is the elevated risk in children with mental retardation. While numerically higher incidences of RRD were observed in patients without primary IOL implantation and in bilaterally operated eyes compared with their counterparts, these differences did not reach statistical significance in this meta‐analysis. These findings should inform surgical decision‐making and postoperative monitoring protocols. We recommend more rigorous follow‐up schedules for high‐risk groups, especially children with mental retardation who showed an 11% incidence of retinal detachment. Future prospective studies should focus on developing targeted preventive strategies for these high‐risk groups and further investigating the factors contributing to RD risk in different surgical contexts, considering the substantial heterogeneity observed in some subgroups. Additionally, standardised reporting of complications in paediatric cataract surgery would facilitate more comprehensive analyses in the future.

## AUTHOR CONTRIBUTIONS

All authors attest that they meet the current ICMJE criteria for authorship.

## FUNDING INFORMATION

The publication of this article was funded by Qatar National Library.

## Supporting information


**Table S1:** Search strategy.
**Table S2:** Detailed inclusion/exclusion criteria for individual studies.
**Table S3:** Quality assessment using Newcastle‐Ottawa scale (NOS).
**Table S4:** Sensitivity analysis.
**Table S5:** Funnel plots of all included studies.
