# Supplementary material for: Incidence and risk factors of rhegmatogenous retinal detachment following paediatric cataract surgery: A systematic review and meta‐analysis
Source: Acta Ophthalmol. 2025 Jun 4;103(7):764–75. doi: 10.1111/aos.17533 (PMC12531611; doi:10.1111/aos.17533)
Supplement: Supplementary file 1 — Table S1: Search strategy. Table S2: Detailed inclusion/exclusion criteria for individual studies. Table S3: Quality assessment using Newcastle‐Ottawa scale (NOS). Table S4: Sensitivity analysis. Table S5: Funnel plots of all included studies. [file AOS-103-764-s001.docx]

**Supplementary Material**

**Table S1:** Search Strategy.

| **Database** | **Search Strategy** | **Results** |
| --- | --- | --- |
| PubMed | ((((((((((pediatric) OR (children)) OR (infant*)) OR (adolescent*)) AND (Cataract surgery)) OR (Cataract Extraction)) OR (Phaco*)) AND (retinal detachment)) OR (Retinal tears)) OR (holes)) OR (breaks) ((((((((((pediatric) OR (children)) OR (infant*)) OR (adolescent*)) AND (Cataract surgery)) OR (Cataract Extraction)) OR (Phaco*)) AND (retinal detachment)) OR (Retinal tears)) OR (holes)) OR (breaks) | 2260 |
| Google Scholar | ((((((((((pediatric) OR (children)) OR (infant*)) OR (adolescent*)) AND (Cataract surgery)) OR (Cataract Extraction)) OR (Phaco*)) AND (retinal detachment)) OR (Retinal tears)) OR (holes)) OR (breaks) | 200 |
| Scopus | ((((((((((pediatric) OR (children)) OR (infant*)) OR (adolescent*)) AND (Cataract surgery)) OR (Cataract Extraction)) OR (Phaco*)) AND (retinal detachment)) OR (Retinal tears)) OR (holes)) OR (breaks) | 146 |
| Cochrane Library | ((((((((((pediatric) OR (children)) OR (infant*)) OR (adolescent*)) AND (Cataract surgery)) OR (Cataract Extraction)) OR (Phaco*)) AND (retinal detachment)) OR (Retinal tears)) OR (holes)) OR (breaks) | 29 |

##

## Table S2: Detailed Inclusion/Exclusion Criteria for Individual Studies

| **Study** | **Inclusion Criteria** | **Exclusion Criteria** | **Minimum Follow-up Period** |
| --- | --- | --- | --- |
| Sabr et al. 2024 | - Children <20 years of age- Congenital cataract diagnosis- Cataract surgery before age 6 years- Complete clinical records available | - Previous ocular surgery- Additional congenital anomalies affecting the eye- Systemic diseases affecting the eye- Incomplete follow-up data | At least 2 years |
| Yen et al. 2023 | - Children <13 years without baseline glaucoma- Lensectomy with primary IOL implantation- Surgery within previous 45 days | - Traumatic cataract- Preexisting glaucoma- Aphakic eyes undergoing secondary IOL surgery | 5 years (or to last follow-up) |
| Oke et al. 2022 | - Patients ≤16 years who underwent cataract surgery- Continuous enrollment prior to cataract surgery (≥6 months for children aged ≥1 year) | - History of RD- RD repair- Traumatic cataract- Spherophakia- Ectopia lentis | Not specified |
| Ngoy et al. 2020 | - Children with bilateral cataract aged 0.4 to 17 years- Surgery between 2001 and 2016 | - Systemic anomalies- Traumatic cataracts- Anterior or posterior segment digenesis- Lens subluxation- Aniridia | At least 24 months |
| Koch et al. 2019 | - Children with bilateral cataract- Cataract surgery with IOL implantation between 1997 and 2007 | - Systemic anomalies- Traumatic cataracts- Anterior or posterior segment digenesis- Lens subluxation- Aniridia | At least 24 months |
| Agarkar et al. 2018 | - Children aged <16 years- Underwent primary lens aspiration, posterior capsulorrhexis, and anterior vitrectomy with primary IOL implantation- Surgery between 1996 and 2007 | - Traumatic cataract- Systemic anomalies (Marfan syndrome, Ehlers-Danlos syndrome)- Ocular anomalies (ROP, PHPV, coloboma, disc anomalies, uveitis, etc.)- Pre-existing glaucoma- Pre-existing RD | Not specific minimum, median follow-up was 66 months |
| Giles et al. 2016 | - Children aged 5-15 years- Cataract surgery with primary IOL implantation- Surgery between 2006 and 2015 | - Active uveitis- 360-degree posterior synechia- Glaucoma- Mental retardation- Non-cooperation with local anesthesia- No concrete light projection | Mean follow-up of 15.75 ± 3.36 weeks |
| Haargaard et al. 2014 | - Children aged 0-17 years with cataract- Underwent cataract surgery between 1977 and 2005- Cases identified from Danish National Patient Register | - Traumatic cataract- Acquired systemic pathology (e.g., diabetes)- Acquired ocular pathology (e.g., uveitis)- Persistent fetal vasculature (PFV)- Retinopathy of prematurity (ROP) | Median follow-up was 6.8 years |
| Rabiah et al. 2005 | - Children aged ≤16 years undergoing lensectomy- Surgery between 1983 and 1996- No other ocular abnormalities except microcornea | - Traumatic cataract- Pre-existing ocular or systemic anomalies- History of ocular trauma or surgery- Primary IOL implantation | Minimum of 2 years |
| **RD**: Retinal Detachment; **IOL**: Intraocular Lens; **ROP**: Retinopathy of Prematurity; **PHPV**: Persistent Hyperplastic Primary Vitreous | | | |

**Table S3:** Quality assessment using Newcastle-Ottawa scale (NOS).

| **Author** | **Year** | **Selection** | | | | **Comparability** | **Outcome/Exposure** | | |  |
| --- | --- | --- | --- | --- | --- | --- | --- | --- | --- | --- |
|  |  | **Adequate definition of patient cases** | **Representativeness of patient cases** | **Selection of controls** | **Definition of controls** | **Control for important or additional factors** | **Ascertainment of exposure** | **The same method of ascertainment for participants** | **Nonresponse rate** | **Total score** |
| Sabr et al. | 2024 | * | * | - | - | - | * | * | * | 5 |
| Yen et al. | 2023 | * | * | - | - | ** | * | * | * | 8 |
| Oke et al. | 2022 | * | * | - | - | - | * | * | * | 5 |
| Ngoy et al. | 2020 | * | * | - | - | - | * | * | * | 5 |
| Koch et al. | 2020 | * | * | - | - | ** | * | * | * | 6 |
| Rabiah et al. | 2019 | * | * | * | * | ** | * | * | * | 6 |
| Agarkar et al. | 2017 | * | - | * | * | ** | * | * | * | 8 |
| Giles et al. | 2016 | * | * | - | - | - | * | - | - | 4 |
|  |  |  |  |  |  |  |  |  |  |  |

**Table S4:** Sensitivity Analysis

| Sensitivity analysis after removing study by Giles et al. and Koch et al. | **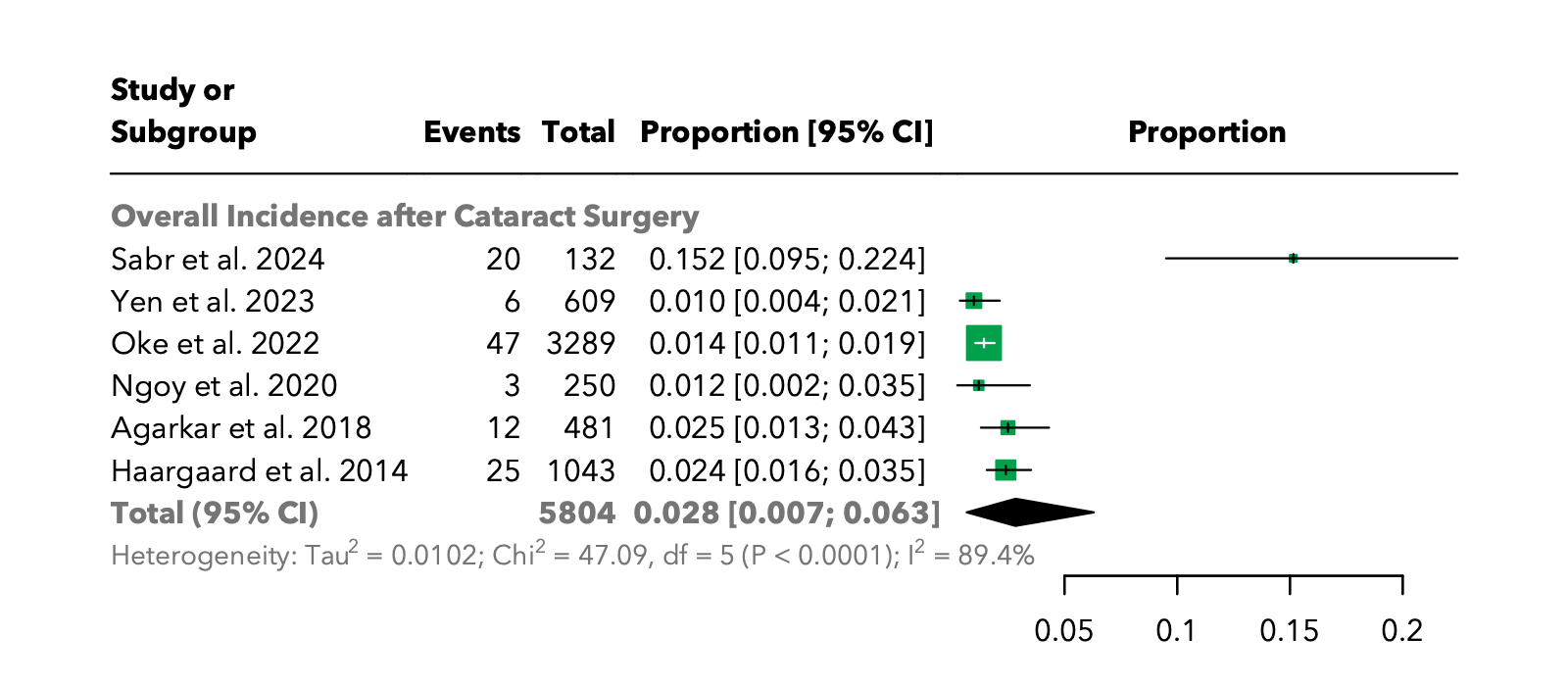** |
| --- | --- |
| L1O analysis of overall incidence of RD after cataract surgery | **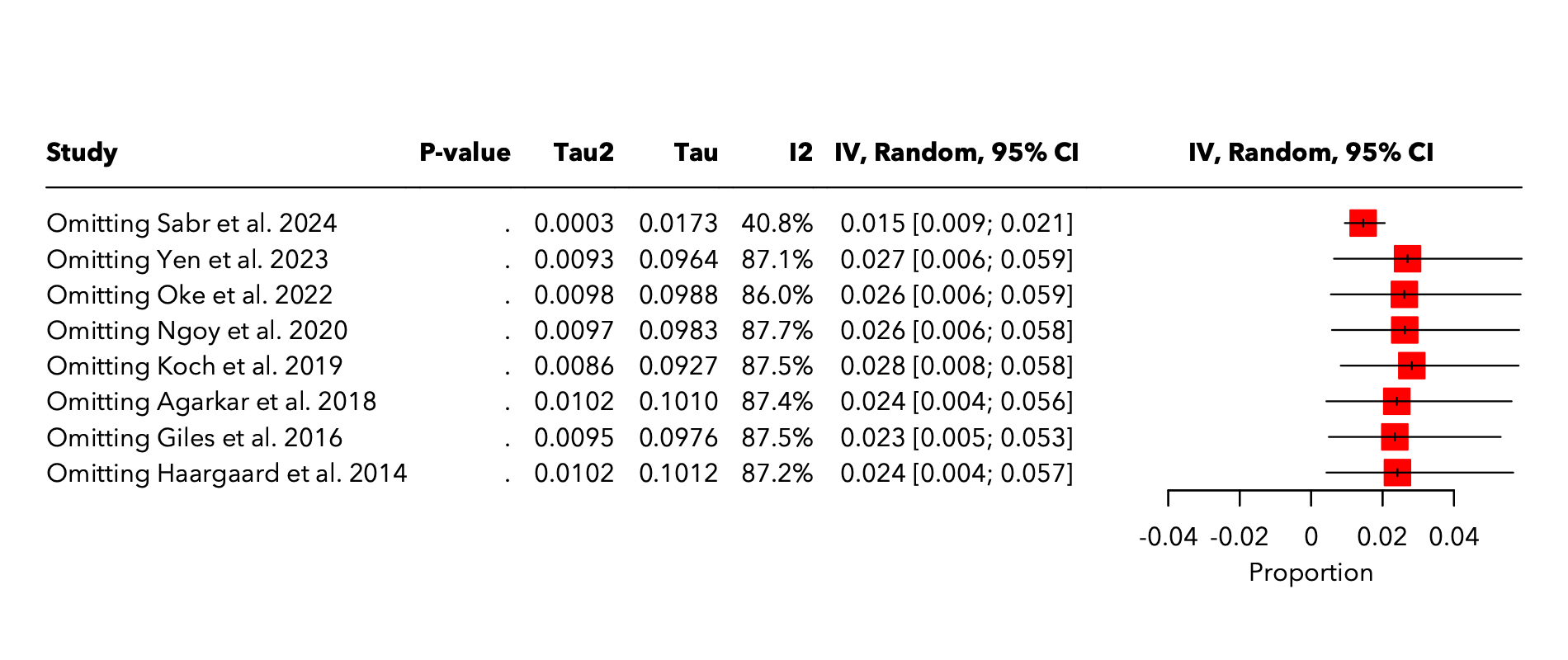** |
| L1O analysis of RD incidence after cataract surgery in patients with Primary IOL Implantation | 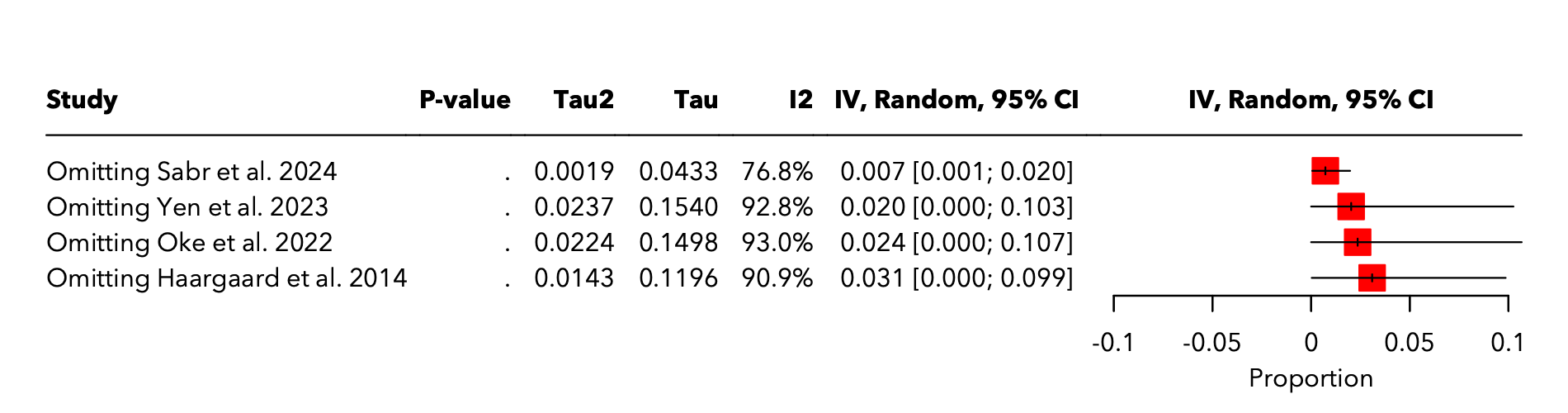 |
| L1O analysis of RD incidence after cataract surgery in patients without Primary IOL Implantation | 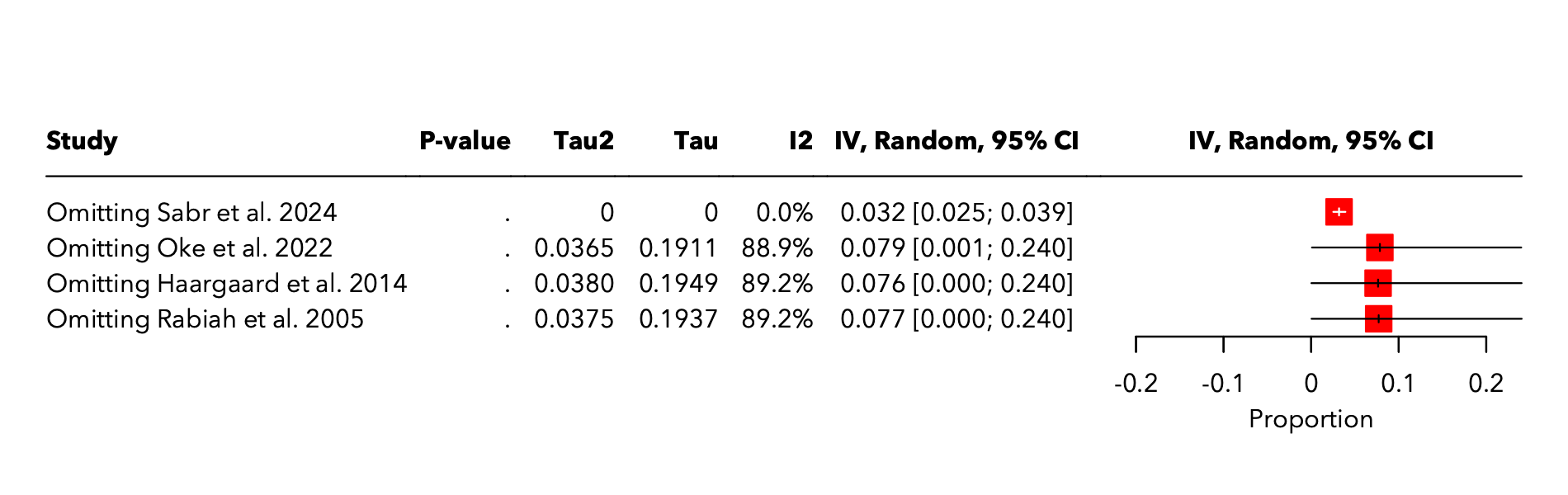 |
| L1O analysis of RD incidence after cataract surgery in bilaterally operated eyes | 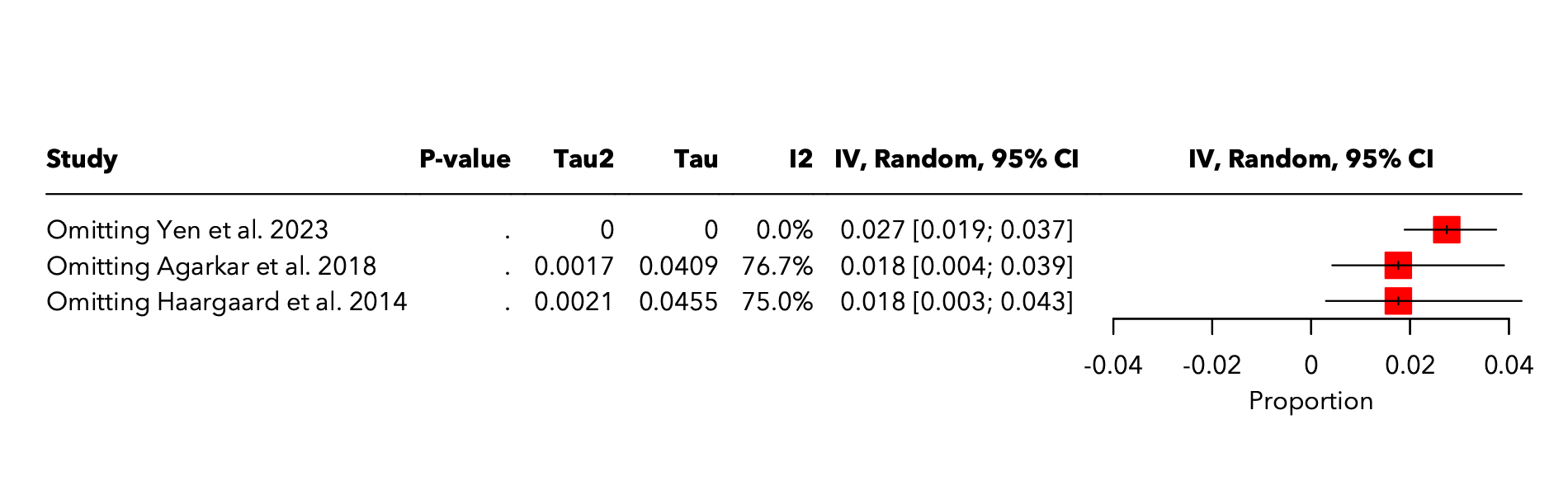 |

**Table S5.** Funnel Plots of All included studies

| Overall Incidence of Retinal Detachment after Cataract Surgery | 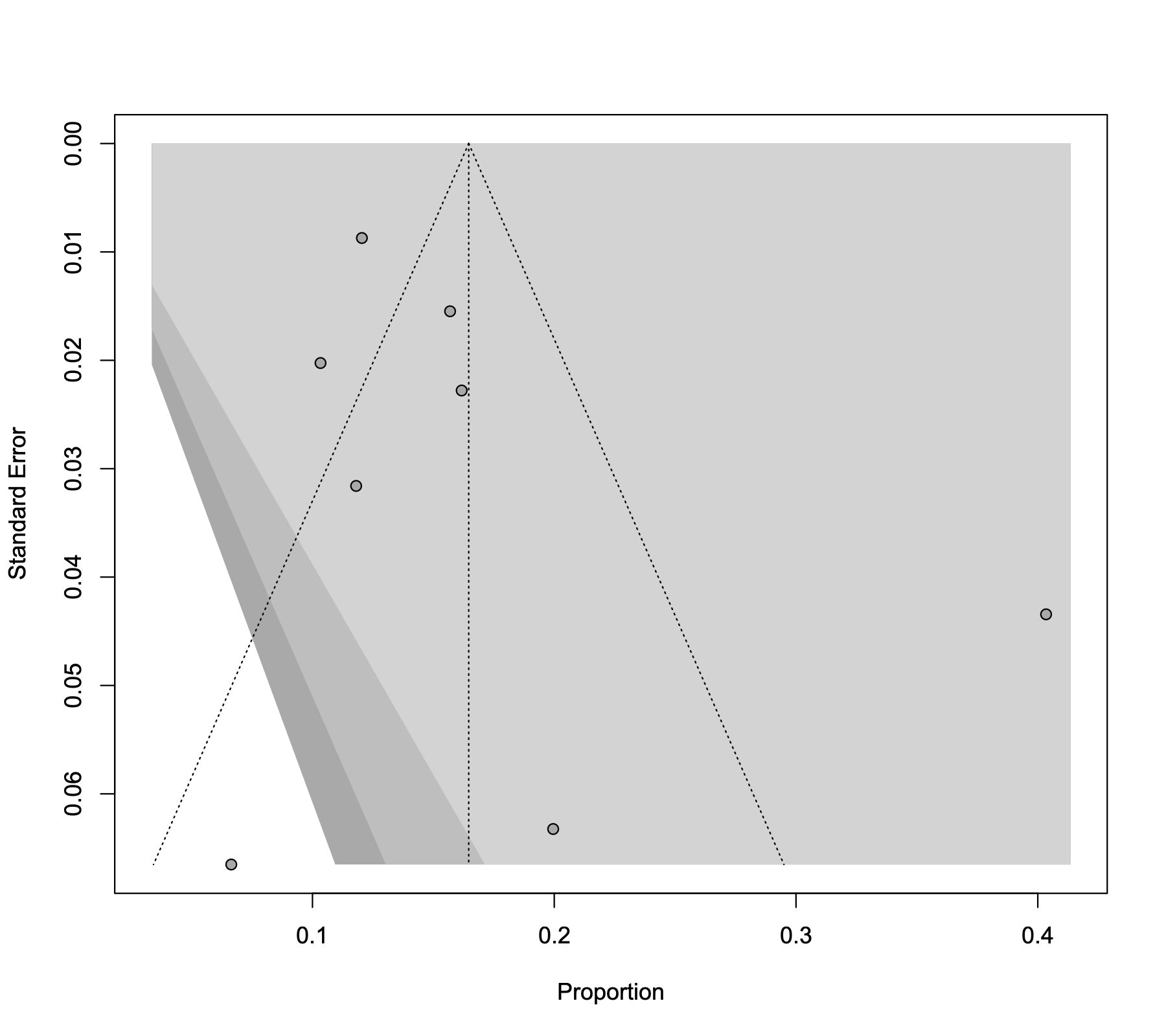 |
| --- | --- |
| Incidence of Retinal Detachment after Cataract Surgery in Patients with and without Primary IOL Implantation | 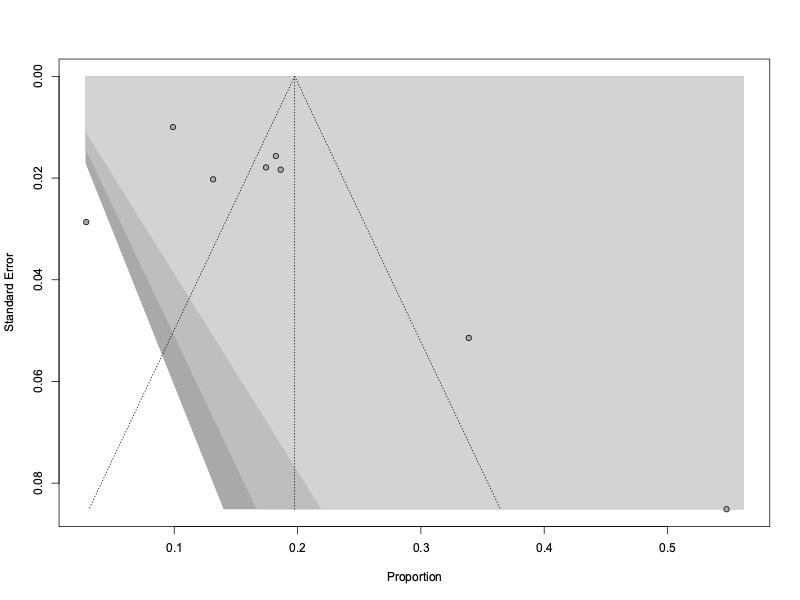 |
| Incidence of Retinal Detachment after Cataract Surgery in Unilaterally and Bilaterally Operated Eyes | 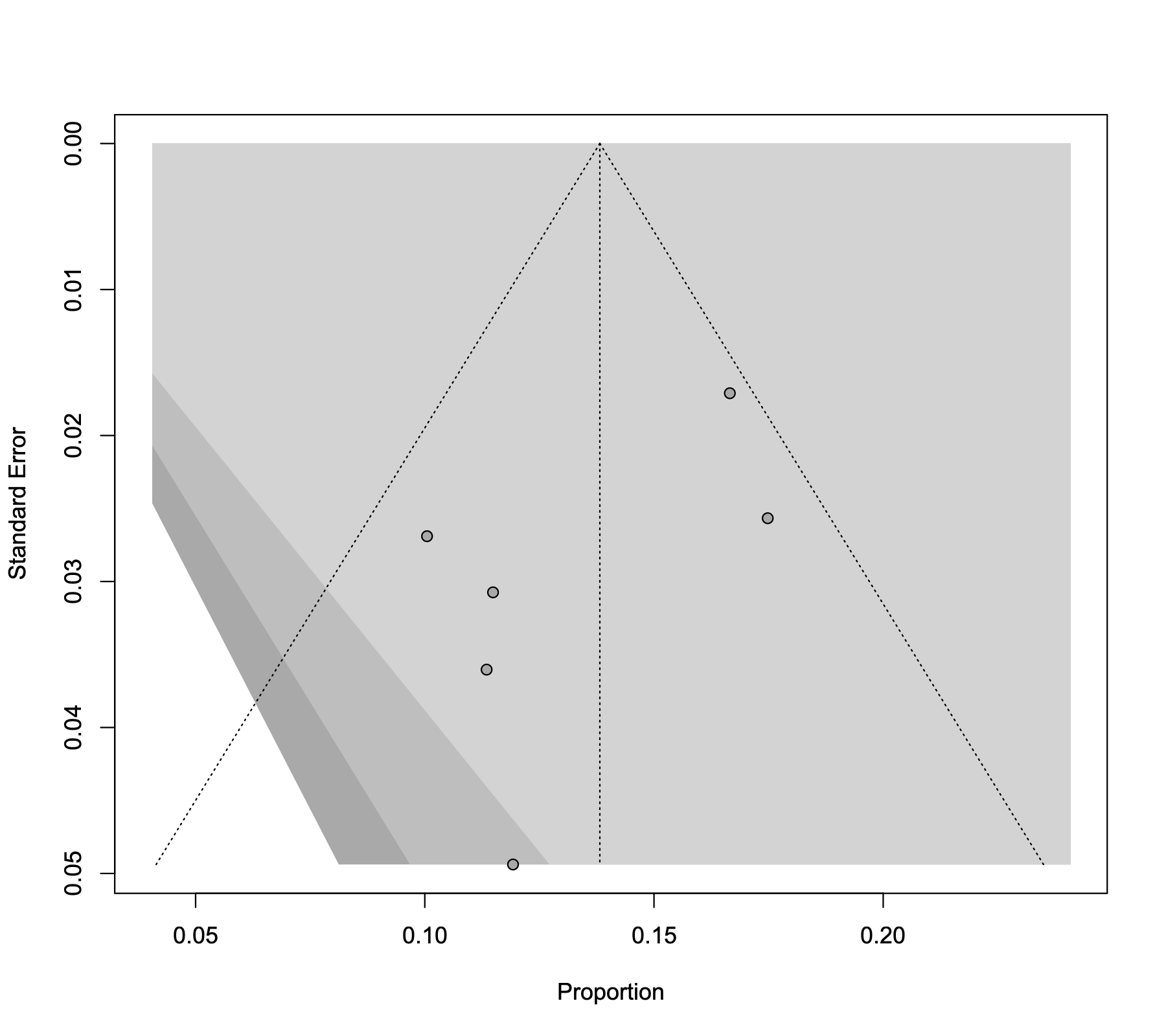 |
| Incidence of Retinal Detachment after Cataract Surgery in Children with Mental Retardation | 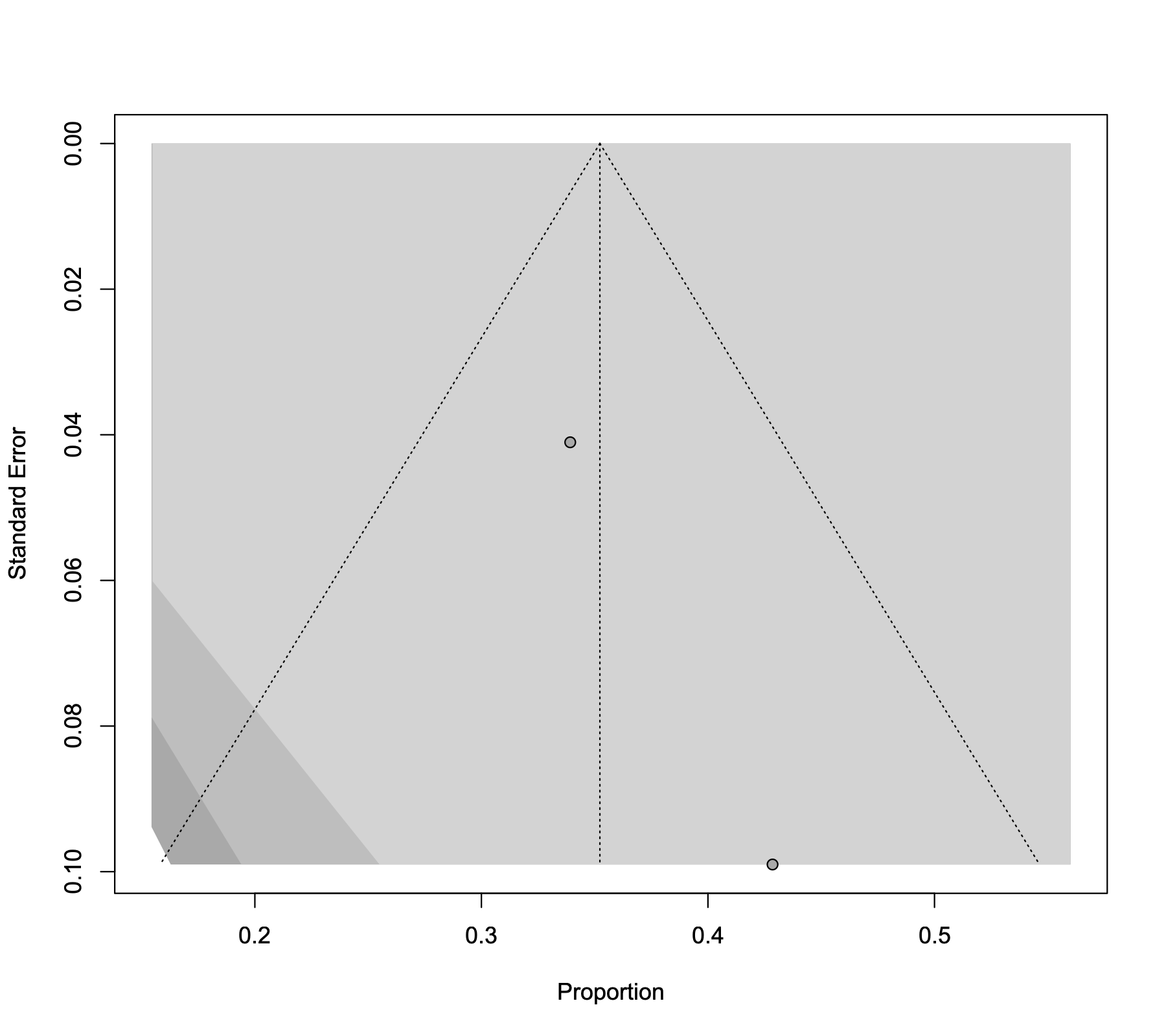 |
